# Supplementary material for: In Vitro Analyses of Spinach-Derived Opioid Peptides, Rubiscolins: Receptor Selectivity and Intracellular Activities through G Protein- and β-Arrestin-Mediated Pathways
Source: Molecules. 2021 Oct 8;26(19):6079. doi: 10.3390/molecules26196079 (PMC8513079; doi:10.3390/molecules26196079)
Supplement: Supplementary file 1 [file molecules-26-06079-s001.zip › molecules-1405994-supplementary.pdf]

Materials

The Fmoc amino acid derivatives were purchased from Watanabe Chemical Ind., Ltd (Hiroshima, Japan). The other reagents were purchased from BLD Pharmatech Ltd. (Shangai, China), Combi-Blocks, Inc. (San Diego, USA), or Watanabe Chemical Ind., Ltd.

Analytical HPLC was performed using a C18 reverse phase column ($5C_{18}$-AR-II, 4.6 I.D. × 150 mm; a COSMOSIL packed column; Nacalai Tesque Inc.) on a Hitachi L-2400 (Hitachi, Ltd., Tokyo, Japan) with a binary solvent system; a linear gradient of CH_3CN in 0.1% aqueous TFA at a flow rate of 1.0 mL/min with detection at 220 nm. Preparative HPLC was carried out using a C18 reversed phase column ($5C_{18}$-AR-II, 10 I.D. × 250 mm; COSMOSIL Packed column, Nacalai Tesque, Inc.) on a Hitachi L-2400 with a binary solvent system; a linear gradient of CH_3CN in 0.1% aqueous TFA at a flow rate of 3 mL/min with detection at 220 nm. The solvents were of HPLC grade. The purified products were identified by ESI-TOF-MS, which was recorded on a JMS-T100LP (JEOL Ltd., Tokyo, Japan) mass spectrometer in the positive detection mode.

Synthesis of rubiscolin-6 (H-Tyr-Pro-Leu-Asp-Leu-Phe-OH)

Rubincolin-6 was synthesized by the standard Fmoc-based solid-phase peptide synthesis using 2-chlorotrityl chloride resin as reported procedure[1,2]. In the protecting groups for the side-chains of Asp and Tyr, OBu*ᵗ* and Bu*ᵗ* were adopted, respectively.

2-Chlorotrityl chloride resin (300 mg, 0.474 mmol) was agitated in dehydrated DMF (5 mL) at room temperature over 1 h. Fmoc-Phe-OH (190 mg, 0.490 mmol) and *N*,*N*-diisopropylethylamine (497 μL, 2.85 mmol) were added. After the mixture was stirred at room temperature for 2 h, the resin was washed with dehydrated DMF (5 mL) five times. Non-reacted trityl chloride group on the resin was capped by reacting with dehydrated methanol (400 μL) in the presence of *N*,*N*-diisopropylethylamine (497 μL) in dehydrated DMF (5 mL) for 1 h. The resin was washed with DMF (5 mL) five times.

Fmoc-Phe-*O*-2-chlorotrityl resin was treated with 20% (v/v) piperidine in DMF (5 mL) to remove the Fmoc group on it. The mixture was stirred for 1 min in hand and 15 min on a stirrer, and then washed with DMF (5 mL) six times. Fmoc-Leu-OH (502 mg, 1.42 mmol, 3 eq.) was coupled by *N*,*N*-diisopropylcarbodiimide (222 μL, 1.42 mmol, 3 eq.)-1-hydroxy-7-azabenzotriazole (192 mg, 1.42 mmol, 3 eq.) method for 90 min in DMF (5 mL). The resin was washed with DMF (5 mL) five times. This reaction was monitored by Kaiser test[3]. The condensation of the corresponding Fmoc amino acid derivatives was successively performed by repeating the same deprotection/coupling procedure. After the peptide chain of rubiscolin-6 was elongated, the N-terminal Fmoc group was deprotected. The

protected peptide resin was washed with methanol (5 mL) five times and diethyl ether (5 mL) twice. The resin was dried enough *in vacuo*. The protected peptide-resin (600 mg) was treated with a mixture of trifluoroacetic acid (TFA, 12.0 mL), H_2O (24.0 μL) and triisopropylsilane (24.0 μL) and was stirred at room temperature for 2 h to cleave the synthetic peptide from the resin with the removal of the protective groups. After the filtration of the mixture with a glass filter, the filtrates were concentrated under the reduced pressure. The excess volume of cold diethyl ether (10 mL) was added to the residues to precipitate the crude peptide. The precipitates were washed with cold diethyl ether (10 mL) three times. This crude peptide was purified by preparative RP-HPLC. Rubiscolin-6 (TFA salt) was obtained as a white amorphous powder (30.2 mg, 6% yield from resin).

Rubiscolin-5 (25.3 mg, 6% yield from resin, white amorphous powder) was synthesized in the same procedure as rubiscolin-6.

Rubiscolin-6 (H-Tyr-Pro-Leu-Asp-Leu-Phe-OH)

ESI-TOF-MS; calcd. for $C_{39}H_{55}N_6O_{10}^+$ $[M+H]^+$: 767.3980, found: 767.3938.

HPLC retention time: 25.2 min (single peak, as depicted below) [column, $5C_{18}$-AR-II (4.6 mm I.D. × 150 mm; a COSMOSIL Packed column); solvent, 0.1% TFA/CH_3CN–H_2O (linear gradient from 20:80 to 40:60 over a period of 40 min at a flow rate of 1 mL/min with detection at 220 nm)], purity >95%, The peaks which were eluted 40 min later derived from the HPLC solvent.

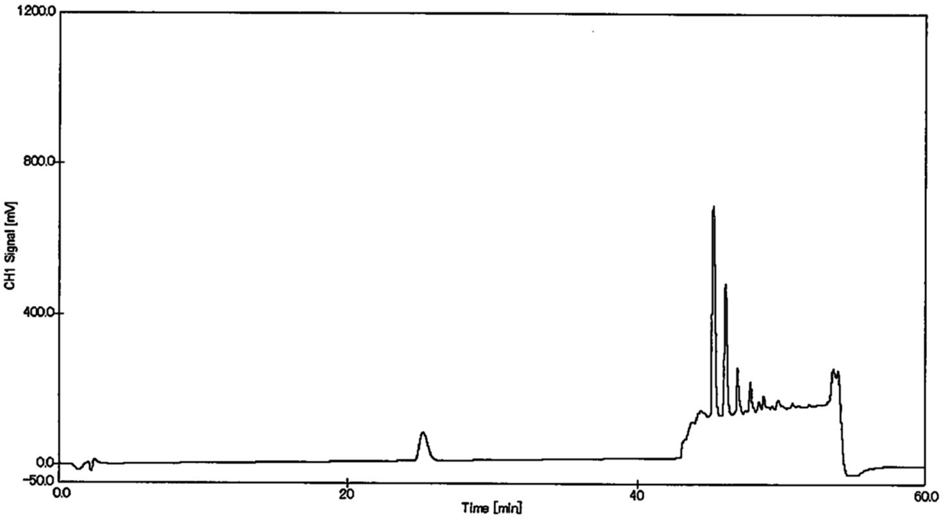

Rubiscolin-5 (H-Tyr-Pro-Leu-Asp-Leu-OH)

ESI-TOF-MS; calcd. for $C_{30}H_{46}N_5O_9^+$ $[M+H]^+$: 620.3296, found: 620.3285.

HPLC retention time: 23.8 min (single peak, as depicted below) column, 5C$_{18}$-AR-II (4.6 mm I.D. × 150 mm; a COSMOSIL Packed column); solvent, 0.1% TFA/CH_3CN–H_2O (linear gradient from 15:85 to 35:65 over a period of 40 min at a flow rate of 1 mL/min with detection at 220 nm)], purity >95%, The peaks which were eluted 40 min later derived from the HPLC solvent.

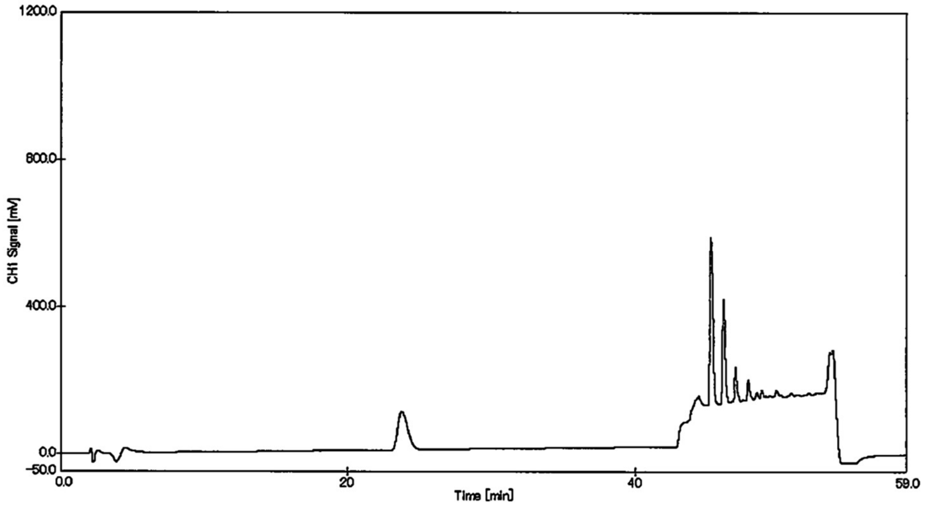

References

1. Mizuguchi, T.; Ohara, N.; Iida, M.; Ninomiya, R.; Wada, S.; Kiso, Y.; Saito, K.; Akaji, K., Evaluation of dimerization-inhibitory activities of cyclic peptides containing a β-hairpin loop sequence of the EGF receptor. *Bioorg. Med. Chem.*, **2012**, *20*, 5730.
2. Toyama, K.; Mizuguchi, T.; Nomura, W.; Tamamura, H., Functional evaluation of fluorescein-labeled derivatives of a peptide inhibitor of the EGF receptor dimerization. *Biorg. Med. Chem.*, **2016**, *24*, 3406.
3. Kaiser, E.; Colescott, R. L.; Bossingner, C. D.; Cook, P. I., Color test for detection of free terminal amino groups in the solid-phase synthesis of peptides. *Anal. Biochem.*, **1970**, *34*, 595.
